# Diagnostic signature composed of seven genes in HIF-1 signaling pathway for preeclampsia

**DOI:** 10.1186/s12884-023-05559-9

**Published:** 2023-04-05

**Authors:** Xun Yang, Ling Yu, Yiling Ding, Mengyuan Yang

**Affiliations:** Department of Obstetrics and Gynecology, The Second Xiangya Hospital, Central South University, Changsha, Hunan Province 410011 China

**Keywords:** Placenta, Microarray, Integrated analysis, Consensus clustering, Logistic regression model, Immune cell infiltration

## Abstract

**Purpose:**

In this study, we explored the relationship of genes in HIF-1 signaling pathway with preeclampsia and establish a logistic regression model for diagnose preeclampsia using bioinformatics analysis.

**Method:**

Two microarray datasets GSE75010 and GSE35574 were downloaded from the Gene Expression Omnibus database, which was using for differential expression analysis. DEGs were performed the Gene Ontology (GO) analysis, Kyoto Encyclopedia of Genes and Genomes (KEGG) pathway enrichment analysis and Gene set enrichment analysis (GSEA). Then we performed unsupervised consensus clustering analysis using genes in HIF-1 signaling pathway, and clinical features and immune cell infiltration were compared between these clusters, as well as the least absolute shrinkage and selection operator (LASSO) method to screened out key genes to constructed logistic regression model, and receiver operating characteristic (ROC) curve was plotted to evaluate the accuracy of the model.

**Results:**

57 DEGs were identified, of which GO, KEGG and analysis GSEA showed DEGs were mostly involved in HIF-1 signaling pathway. Two subtypes were identified of preeclampsia and 7 genes in HIF1-signaling pathway were screened out to establish the logistic regression model for discrimination preeclampsia from controls, of which the AUC are 0.923 and 0.845 in training and validation datasets respectively.

**Conclusion:**

Seven genes (including MKNK1, ARNT, FLT1, SERPINE1, ENO3, LDHA, BCL2) were screen out to build potential diagnostic model of preeclampsia.

## Introduction

Preeclampsia, a disease that affects about 3–5% of all pregnancies worldwide, is one of the major causes of maternal and perinatal mobility and mortality globally [1, 2]. Occurs to pregnant women after 20 gestational weeks, preeclampsia is characterized mostly by hypertension with blood pressure more than 140/90 mmHg, complicates with proteinuria over 300 mg in 24 h [3]. Due to the endothelial disfunction in preeclampsia, extensive organs all over the body can be injured, including the liver, kidney, blood system and brain [4]. As preeclampsia progresses, it turns to be eclampsia, a condition which is life threatening for both gravidas and fetus [5, 6].

As the heterogeneity of physiopathology in preeclampsia, its etiology is not fully elucidated [7]. Previous researchers have found many biomarkers associated with preeclampsia such as sFlt-1 and PLGF, but their reliability to diagnosing preeclampsia is not sufficient, and progress with biomarkers studies remains limited [4, 8]. Consequently, discovering novel biomarkers are necessary for the diagnosis of preeclampsia.

Although the mechanism underlying preeclampsia is still unclear, existing studies demonstrated that inflammation and oxidative stress is an essential part of the physiopathology of preeclampsia [9]. Previous studies have reported that placenta hypoxia is associated with the pathogenesis of preeclampsia, while they mostly focus on hypoxia inducible factor [10, 11]. Few research has explored the relationship between HIF-1 signaling pathway and preeclampsia. It has been researched that some genes of HIF1-sinaling pathway are related to inflammation and oxidative stress, so HIF1-signaling pathway might be involved in preeclampsia. Meanwhile, the results of our analysis showed that HIF-1 signaling pathway is associated with preeclampsia. In this study, we explored the genes of HIF-1 signaling pathway in preeclampsia through bioinformatics method. Firstly, we try to grouping preeclampsia into different subtypes through HIF1-signaling pathways genes. Accordingly, clinical features and immune cell infiltration were compared between different subtypes, of which the results indicated that HIF1-signaling pathway might play a vital part in preeclampsia. Then we screened out seven genes (including MKNK1, ARNT, FLT1, SERPINE1, ENO3, LDHA, BCL2) in HIF1-signaling pathway. It was the first time to use HIF-1 pathway for constructed a diagnostic model of preeclampsia, which could distinguish preeclampsia from controls with a good accuracy.

## Methods

### Data downloading and preprocessing

Two mRNA datasets including GSE75010 [12] and GSE35574 [13] was downloading from the Gene Expression Omnibus database (GEO, https://www.ncbi.nlm.nih.gov/geo/). GSE75010 dataset included 157 placenta samples consisting of 80 placenta samples from preeclampsia patients and 77 placenta samples from control patients. GSE35574 dataset included 94 placenta samples consisting of 35 placenta samples from IUGR patients, 19 placenta samples from preeclampsia patients and 40 placenta samples from control patients. Then we select the 19 preeclampsia and 40 control placenta samples of GSE35574 dataset for analysis. GSE75010 dataset was used as training dataset and GSE35574 dataset was used as the external validation dataset. Firstly, we transformed the probe numbers of the two datasets to gene symbols and remove the null probes using R language. Both of the two datasets were normalized by using Robust Multi-Array Average (RMA) method, and then was log2 transformed using R language. And HIF-1 signaling pathway genes were download from Kyoto Encyclopedia of Genes and Genomes (KEGG) [14]. In GSE75010, preeclampsia was defined as the onset of systolic pressure ≥ 140 mmHg and/or diastolic pressure ≥ 90 mmHg after the 20th week of gestation, accompanied by proteinuria (greater than 300 mg protein/day, or greater ≥ 2 + by dipstick). Patients with diabetes (pre-existing or gestational), sickle cell anemia and/or morbid obesity (BMI ≥ 40) were excluded (Table 1), and all samples came from singleton pregnancies [12]. And in GSE35574, the PE was defined as a sustained (≥ 2 measures 6 h apart) blood pressure elevation (> 140/90 mm Hg) > 20 weeks of gestation with proteinuria defined as a sustained (≥ 2 measures 4 h apart) presence of elevated protein in the urine (> 30 mg/dL or > 1 + on a urine dipstick) [13]. Because all data for this study were obtained from public databases, the study did not require the institutional review board approval.

**Table 1:** Clinical characteristics of GSE75010

Characteristic	Control (n = 77)	PE (n = 80)	p value
Delivery weeks	34 + 3	32 + 5	0.012
Maternal age	33.2	33.2	0.995
Maternal bmi	24.5	26.5	0.026
Maximum diastolic bp	85.3	107	< 0.001
Maximum systolic bp	136	170	< 0.001
Proteinuria	0.45	2.59	< 0.001
Umbilical cord diameter	1.21	1.16	0.38
Mode of delivery			0.053
C-Section	50(64.9%)	64(80.0%)	
Vaginal	27(35.1%)	16(20.0%)	

### Differential expression analysis

Limma packages [15] was using in R language to perform the differential expression analysis between preeclampsia samples and control samples of GSE75010 datasets. Differential expressed genes (DEGs) were considered as significant when the |fold change (FC)| > 1.5 and adjusted P value < 0.05. The visualization of these genes was plotted using “pheatmap” and “ggpuber” package in R.

### Functional Enrichment analysis of DEGs

Functional enrichment analysis were performed Gene Ontology (GO) analysis and Kyoto Encyclopedia of Genes and Genomes (KEGG) [14] pathway enrichment analysis and Gene set enrichment analysis (GSEA) was also performed using “clusterProfiler” in R language [16]. P value < 0.05 was dimmed as significant. The results of these analysis were plotted via “ggplot2” package in R.

### Analysis of unsupervised consensus clustering and immune cell infiltration

Unsupervised consensus clustering analysis was performed in the 80 placenta samples from preeclampsia patients in GSE75010 to elucidate the relationship between genes in HIF-1 signaling pathway and preeclampsia subtypes using “ConsensusClusterPlus” package [17] in R language with hierarchical clustering, pearson distance, maxK = 10, reps = 1000, pItem = 0.8, and pFeature = 0.8. The clinical features between these clusters were compared after consensus clustering with Wilcoxon rank sum tests. Moreover, immune cell infiltration analysis was performed by Cibersort algorithm using “IOBR” package [18] with perm = 1000 and QN = T to elucidate the composition of immune cells between these clusters.

### Construction of logistic regression model

The least absolute shrinkage and selection operator (LASSO) method was performed using “glmnet” package [19] with family = binomial, nlambda = 1000 and alpha = 1 in R language to screen out genes to construct logistic regression model. Then the genes were using to construct logistic regression model in GSE75010 training dataset using package “nnet” [20]. Then, receiver operating characteristic (ROC) curve using package “ROCR” [21] was plotted to evaluate the reliability of the logistic regression model. Furthermore, the GSE35574 dataset was used as the external validation dataset.

## Results

### Differential expressed genes in GSE75010 dataset

**Fig. 1:**
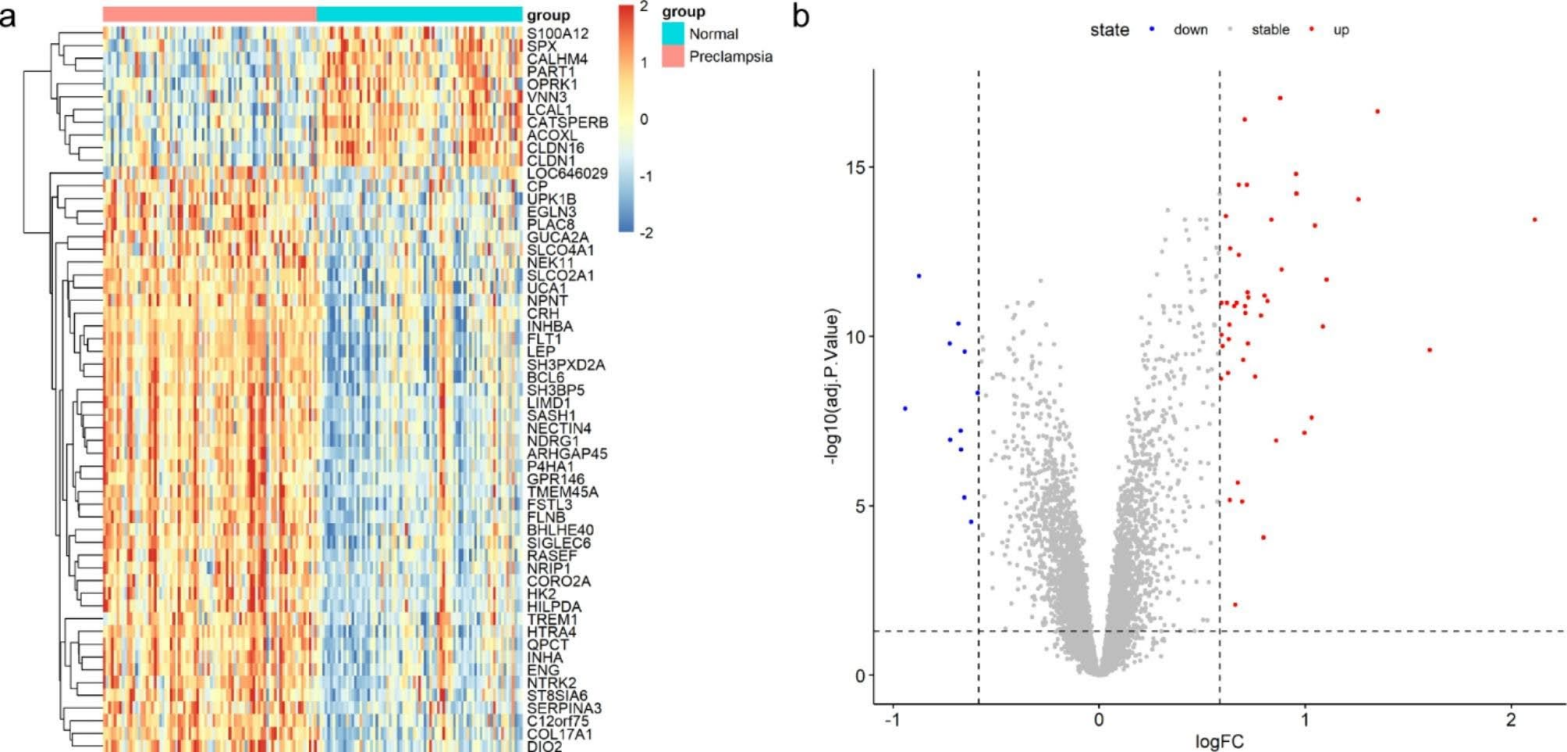
The DEGs between preeclampsia and control placenta of GSE75010 datasets. (**a**) The heatmap of the 57 DEGs. The horizontal axis represents samples and the vertical axis represents genes. The color indicates the gene expression values. (**b**) The volcano plot. Each point represents a gene, and red ones represent upregulated genes, while blue ones represent downregulated gene

Differential expression analysis was performed between preeclampsia samples and control samples in GSE75010 datasets. 57 differential expressed genes are screen out, which are composed of 46 upregulated genes and 11 downregulated genes (Fig. 1).

### Functional Enrichment Analysis

**Fig. 2:**
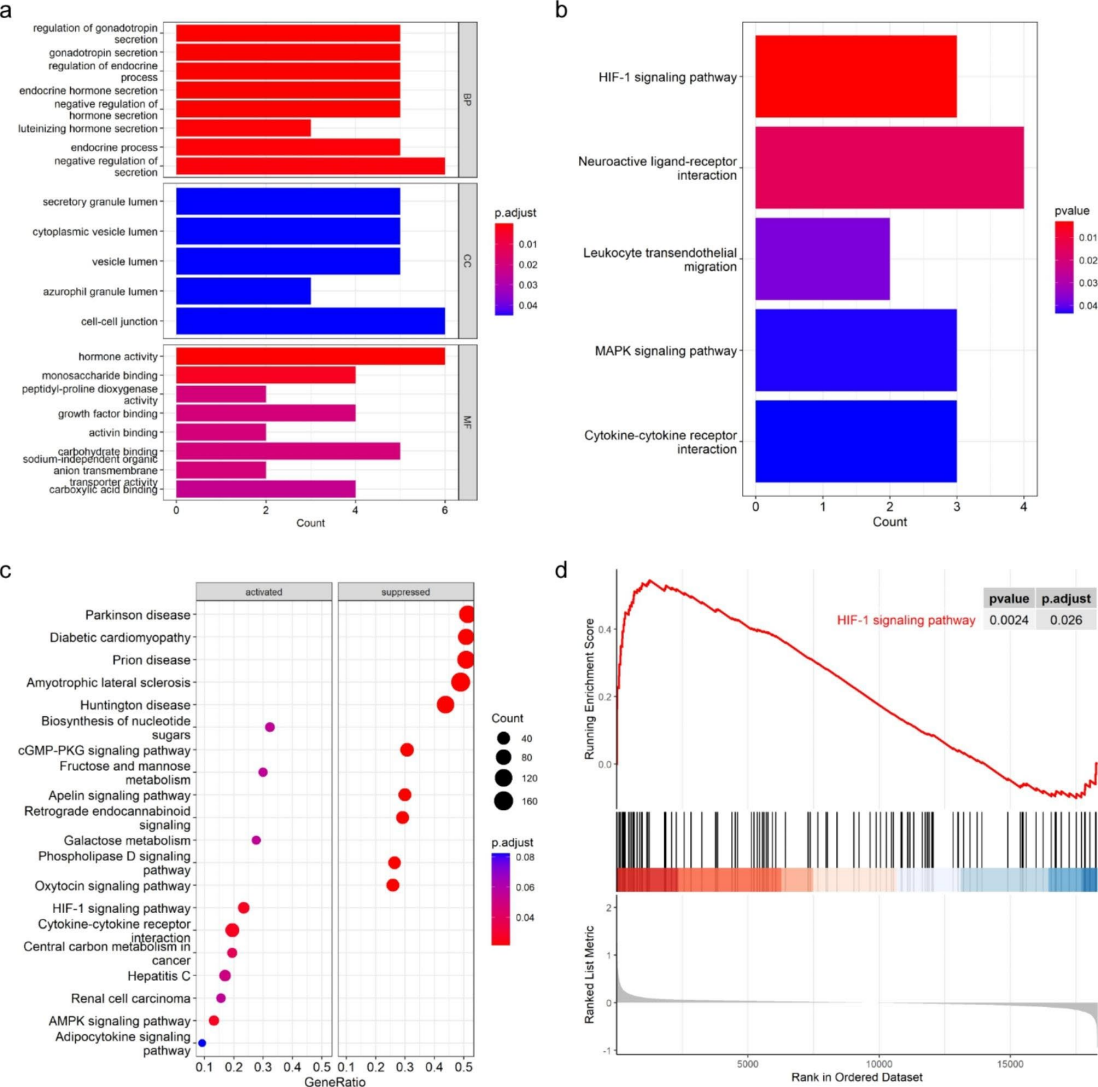
The results of the Functional Enrichment Analysis in GSE75010 datasets. **a**, **b**. The Gene Ontology (GO) analysis and the Kyoto Encyclopedia of Genes and Genomes (KEGG) pathway enrichment analysis. The horizontal axis is gene counts and the vertical axis is pathways. **c**. The Gene set enrichment analysis (GSEA). The horizontal axis is gene ratio and the vertical axis is pathways. The activated part is the GSEA results of upregulated genes and the suppressed part is the GSEA results of the downregulated genes. **d**. The HIF-1 signaling pathway enriched in GSE75010, which shows significant difference between preeclampsia and healthy samples

To illustrate the function of DEGs, we performed GO and KEGG enrichment analyses and GSEA. The outcomes of GO analyses showed that DEGs were most significantly enriched in biological process (BP) such as “regulation of gonadotropin secretion”, in cellular component (CC) such as “secretory granule lumen” and in molecular function (MF) such as “hormone activity”. The result of KEGG analyses showed that DEGs were mainly enriched in HIF-1 signaling pathway and neuroactive ligand-receptor interaction. And then, the results of GSEA also enriched in HIF-1 signaling pathway, which reveal that HIF-1 signaling pathway may play an important role in preeclampsia (Fig. 2).

### Identification of HIF subtypes of preeclampsia

**Fig. 3:**
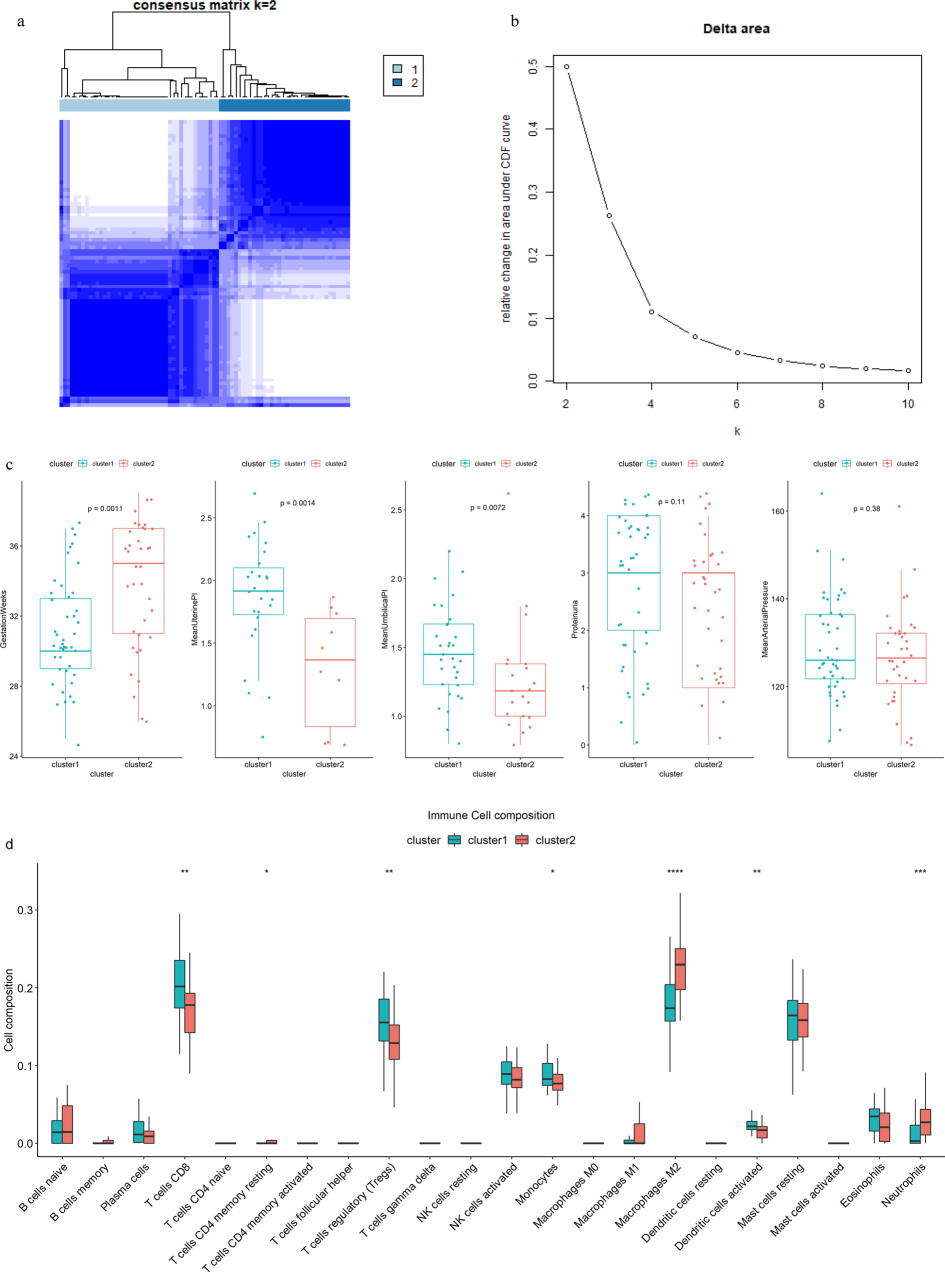
The results of consensus clustering analysis and the comparison of the features between the two cluster. (**a**) Consensus clustering matrix when k = 2. (**b**) The delta area plot of consensus clustering, which indicates the best k value is 2. (**c**) The comparison of the clinical features between the two clusters. (**d**) The immune infiltration levels in the two clusters (*p < 0.05, **p < 0.01, ***p < 0.001 and ****p < 0.0001)

According to the expression level of genes in HIF-1 signaling pathway, the 80 preeclampsia patients in dataset GSE75010 were divided into two subtypes: Cluster1 (n = 44), and Cluster2 (n = 36) (Fig. 3a, b). Then the clinical features between two clusters were compared, the gestation weeks, mean uterine pulsatility index (PI), and mean umbilical pulsatility index (PI) of cluster1 significantly different with cluster2, while there are no significantly different with proteinuria and mean arterial pressure within two classes (Fig. 3c). From the results of the clinical features comparison between the two clusters, we could draw the conclusion that cluster1 might have worse prognosis than cluster2. Moreover, using the Cibersort algorithm, we analyzed the immune cell infiltration in the two clusters, which showed significant difference in T cells CD8, T cells CD4 memory resting, T cells regulatory (Tregs), Monocytes, Macrophages M2, Dendritic cells activated and Neutrophils (Fig. 3d).

### Construction and validation of the diagnostic genes signature

**Fig. 4:**
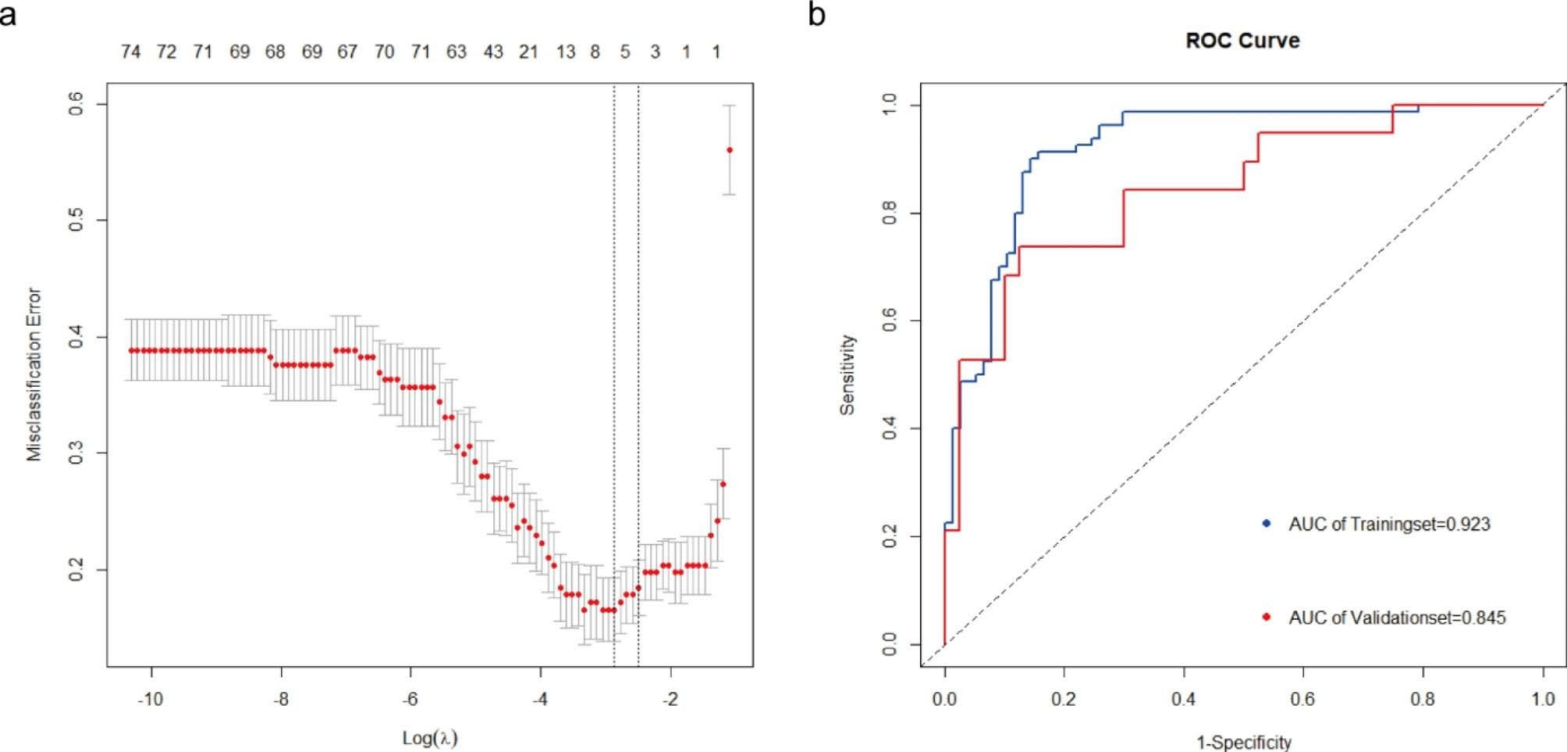
(**a**) The genes selection using lasso method. (**b**) The ROC curve of training datasets GSE75010 and validation datasets GSE35574

Then the LASSO regression model was employed to screen for the most robust biomarkers to create an HIF-1 signaling pathway genes-related diagnostic signature in the training set GSE75010 (Fig. 4a). Seven genes were identified to construct the diagnostic signature: MKNK1, ARNT, FLT1, SERPINE1, ENO3, LDHA, BCL2. And Logistic regression model was constructed with the seven genes in the training set GSE75010. To confirm the accuracy of the model, we plot the ROC curves of the model in two datasets. The area under curve (AUC) values in the training set GSE75010 and validation set GSE35574 were 0.923 and 0.845, respectively (Fig. 4b).

## Discussion

Preeclampsia is a heterogeneous, pregnancy-specific syndrome clinically characterized by the development of hypertension and proteinuria, as well as the leading cause of maternal and perinatal mortality and morbidity. Although the etiology of preeclampsia remains largely unclear, the main hypotheses strongly rely on disturbed placental function pregnancy. As placenta is the key organ involved in preeclampsia, analysis of genes expressed in placenta become a vital way to explore the molecular mechanism underlying preeclampsia, contributing to the discovery of potential biomarkers for diagnostic and therapeutic targets [1, 4].

We analyzed the gene expression profiles of placenta samples between preeclampsia and controls, and 57 differentially expressed genes were identified. Consistent with published data, the results of enrichment of these DEGs confirm their involvement in the development of preeclampsia, such as HIF1-signaling pathway, MAPK signaling pathway, cytokine-cytokine receptor interaction, which suggest that Inflammation and oxidative stress is important in preeclampsia [22, 23]. It has been widely accepted that Inflammation and oxidative stress are vital processes concerned with placental ischemia and hypoxia in the development of preeclampsia, and part of genes in HIF1-signaling pathway are closely related to inflammation and oxidative stress. Precious study indicated that p38 MAPK plays a vital role in PE progression [24]. It has been reported HIF-1β is essential for the elevated production of sFLT1 in the hypoxic trophoblasts [10]. Based on these reports, along with our results of the enrichment analysis and subtyping analysis, HIF1-signaling pathway might play a part in the pathogenesis of preeclampsia.

Then we perform consensus clustering analysis of genes in HIF1-signaling pathways, and two clusters were divided. Clinical manifestations were compared between the two clusters, the results of which showed that the cluster1 has significantly less gestation weeks than cluster2 did, at the same time, the mean uterine pulsatility index (PI) and mean umbilical pulsatility index (PI) in cluster1 were significantly higher than in cluster2, which indicated that cluster1 might have a worse prognosis than cluster2. Additionally, the composition of 22 immune cells were calculated, and 7 immune cells, namely, T cells CD8, T cells CD4 memory resting, T cells regulatory (Tregs), monocytes, macrophages M2, dendritic cells activated and neutrophils, were significantly different between the two clusters. Previous studies had found that T cells CD8 are crucial for immune tolerance and immunity, and infiltration of T cells CD8 into the placental villous tissue was a feature in abnormal placenta of preeclampsia [25, 26]. T cells regulatory (Tregs), a subset of suppressor CD4(+) T cells, play a vital role in the maintaining of immune balance of maternal-fetus interface, which are involved in the development of preeclampsia [27, 28]. Monocytes are found in most human tissues, which can differentiate to macrophages such as macrophages M1 and macrophages M2. Macrophages M1 and Macrophages M2 participate in the proinflammatory and anti-inflammatory activity respectively, what`s more, their alteration of polarity is associated with preeclampsia [29]. Neutrophils has been reported to produce massive reactive oxygen species (ROS) in the development of preeclampsia [30]. On the whole, immune cell infiltration plays an important role in preeclampsia, and the differences between the two HIF-1 associated clusters indicated that HIF-1 signaling pathway might have a crucial role in the pathophysiology of preeclampsia.

Moreover, seven genes in HIF-1 signaling pathways were screened out with LASSO to construct the logistic regression model including MKNK1, ARNT, FLT1, SERPINE1, ENO3, LDHA, BCL2. MKNK1 was found significantly increased in FGR-affected placenta [31], while its function in preeclampsia remained to be investigated. Previous study has reported that HIF-1 Beta, encoded by ARNT, is associated with placental morphogenesis, angiogenesis, and cell differentiation [32]. FLT1 encodes Fms-related tyrosine kinase 1 (FLT1 or VEGFR1), which is related to reactive oxygen species, and sFlt1, the soluble form of FLT1, is widely used for diagnosis and management in preeclampsia with placental growth factor (PIGF) [4]. SERPINE1 encodes PAI1, which is reported to be an inhibitor of trophoblast migration and invasion [33]. B cell lymphoma 2 (Bcl2) is an antiapoptotic marker which is found lower in preeclampsia placenta than health placenta, while the role BCL2 in preeclampsia needs further research [34]. However, the contribution of ENO3 and LDHA to preeclampsia is still unclear. Based on these seven genes, we constructed a diagnostic model with AUC 0.923 and 0.845 in training dataset and validation dataset, respectively, which means a good performance in distinguishing preeclampsia and healthy pregnancy, and these genes might be potential biomarkers associated with the occurrence and development of preeclampsia. In addition, More attention should be paid to the role of these genes in the physiopathology of preeclampsia. However, because our analysis is based on public databases, further experimental studies are needed to validate the seven genes of the result of this study.

## Conclusion

In summary, our study identified MKNK1, ARNT, FLT1, SERPINE1, ENO3, LDHA, BCL2 out of HIF1-signaling pathway as novel diagnostic biomarkers for preeclampsia patients, and a diagnostic signature based on these genes is constructed for preeclampsia.

## Data Availability

The datasets analyses during the current study are available in the Gene Expression Omnibus database (GEO, https://www.ncbi.nlm.nih.gov/geo/), and the accession numbers for the data sets were GSE75010 and GSE35574.

## References

[1] Chappell LC, Cluver CA, Kingdom J, Tong S (2021). Pre-eclampsia. Lancet.

[2] Ma’ayeh M, Costantine MM (2020). Prevention of preeclampsia. Semin Fetal Neonatal Med.

[3] Poon LC, Shennan A, Hyett JA, Kapur A, Hadar E, Divakar H, McAuliffe F, da Silva Costa F, von Dadelszen P, McIntyre HD, Kihara AB, Di Renzo GC, Romero R, D’Alton M, Berghella V, Nicolaides KH, Hod M (2019). The International Federation of Gynecology and Obstetrics (FIGO) initiative on pre-eclampsia: a pragmatic guide for first-trimester screening and prevention. Int J Gynaecol Obstet.

[4] MacDonald TM, Walker SP, Hannan NJ, Tong S, Kaitu’u-Lino TJ (2022). Clinical tools and biomarkers to predict preeclampsia. EBioMedicine.

[5] Abalos E, Cuesta C, Grosso AL, Chou D, Say L (2013). Global and regional estimates of preeclampsia and eclampsia: a systematic review. Eur J Obstet Gynecol Reprod Biol.

[6] Phipps EA, Thadhani R, Benzing T, Karumanchi SA (2019). Pre-eclampsia: pathogenesis, novel diagnostics and therapies. Nat Rev Nephrol.

[7] Dymara-Konopka W, Laskowska M, Blazewicz A (2018). Angiogenic imbalance as a contributor of Preeclampsia. Curr Pharm Biotechnol.

[8] Bastek JA, Elovitz MA (2013). The role and challenges of biomarkers in spontaneous preterm birth and preeclampsia. Fertil Steril.

[9] Welch BM, McNell EE, Edin ML, Ferguson KK. Inflammation and oxidative stress as mediators of the impacts of environmental exposures on human pregnancy: evidence from oxylipins. Pharmacol Ther. 2022;108181. 10.1016/j.pharmthera.2022.108181. 10.1016/j.pharmthera.2022.108181 PMC9525454 35367517

[10] Sasagawa T, Nagamatsu T, Yanagisawa M, Fujii T, Shibuya M. Hypoxia-inducible factor-1beta is essential for upregulation of the hypoxia-induced FLT1 gene in placental trophoblasts. Mol Hum Reprod. 2021;27. 10.1093/molehr/gaab065. 10.1093/molehr/gaab065 PMC8633902 34665260

[11] Colson A, Depoix CL, Baldin P, Hubinont C, Sonveaux P, Debieve F (2020). Hypoxia-inducible factor 2 alpha impairs human cytotrophoblast syncytialization: new insights into placental dysfunction and fetal growth restriction. FASEB J.

[12] Leavey K, Benton SJ, Grynspan D, Kingdom JC, Bainbridge SA, Cox BJ (2016). Unsupervised placental gene expression profiling identifies clinically relevant subclasses of human preeclampsia. Hypertension.

[13] Guo L, Tsai SQ, Hardison NE, James AH, Motsinger-Reif AA, Thames B, Stone EA, Deng C, Piedrahita JA (2013). Differentially expressed microRNAs and affected biological pathways revealed by modulated modularity clustering (MMC) analysis of human preeclamptic and IUGR placentas. Placenta.

[14] Kanehisa M, Goto S (2000). KEGG: kyoto encyclopedia of genes and genomes. Nucleic Acids Res.

[15] Ritchie ME, Phipson B, Wu D, Hu Y, Law CW, Shi W, Smyth GK (2015). Limma powers differential expression analyses for RNA-sequencing and microarray studies. Nucleic Acids Res.

[16] Yu G, Wang LG, Han Y, He QY (2012). clusterProfiler: an R package for comparing biological themes among gene clusters. OMICS.

[17] Wilkerson MD, Hayes DN (2010). ConsensusClusterPlus: a class discovery tool with confidence assessments and item tracking. Bioinformatics.

[18] Zeng D, Ye Z, Shen R, Yu G, Wu J, Xiong Y, Zhou R, Qiu W, Huang N, Sun L, Li X, Bin J, Liao Y, Shi M, Liao W (2021). IOBR: Multi-Omics Immuno-Oncology Biological Research to Decode Tumor Microenvironment and Signatures. Front Immunol.

[19] Friedman J, Hastie T, Tibshirani R (2010). Regularization Paths for generalized Linear Models via Coordinate Descent. J Stat Softw.

[20] Beck MW (2018). NeuralNetTools: visualization and analysis tools for neural networks. J Stat Softw.

[21] Sing T, Sander O, Beerenwinkel N, Lengauer T (2005). ROCR: visualizing classifier performance in R. Bioinformatics.

[22] Yang MY, Ji MH, Shen T, Lei L. (2022) Integrated Analysis Identifies Four Genes as Novel Diagnostic Biomarkers Which Correlate with Immune Infiltration in Preeclampsia. J Immunol Res 2022:2373694. 10.1155/2022/2373694 10.1155/2022/2373694 PMC9071854 35528613

[23] Liu Y, Wang Z, Zhao L (2021). A potential three-gene-based diagnostic signature for hypertension in pregnancy. Int J Gen Med.

[24] Lu F, Gong H, Lei H, Li J (2022). Downregulation of cathepsin C alleviates endothelial cell dysfunction by suppressing p38 MAPK/NF-kappaB pathway in preeclampsia. Bioengineered.

[25] Lager S, Sovio U, Eddershaw E, van der Linden MW, Yazar C, Cook E, Happerfield L, Jessop FA, Sebire NJ, Charnock-Jones DS, Smith GCS (2020). Abnormal placental CD8(+) T-cell infiltration is a feature of fetal growth restriction and pre-eclampsia. J Physiol.

[26] van der Zwan A, Bi K, Norwitz ER, Crespo AC, Claas FHJ, Strominger JL, Tilburgs T (2018). Mixed signature of activation and dysfunction allows human decidual CD8(+) T cells to provide both tolerance and immunity. Proc Natl Acad Sci U S A.

[27] Ding H, Dai Y, Lei Y, Wang Z, Liu D, Li R, Shen L, Gu N, Zheng M, Zhu X, Zhao G, Hu Y (2019). Upregulation of CD81 in trophoblasts induces an imbalance of Treg/Th17 cells by promoting IL-6 expression in preeclampsia. Cell Mol Immunol.

[28] La Rocca C, Carbone F, Longobardi S, Matarese G (2014). The immunology of pregnancy: regulatory T cells control maternal immune tolerance toward the fetus. Immunol Lett.

[29] Jena MK, Nayak N, Chen K, Nayak NR (2019). Role of macrophages in pregnancy and related complications. Arch Immunol Ther Exp (Warsz).

[30] Liu D, Li Q, Ding H, Zhao G, Wang Z, Cao C, Dai Y, Zheng M, Zhu X, Wu Q, Wang Y, Duan H, Tang H, Lu X, Hou Y, Hu Y (2021). Placenta-derived IL-32beta activates neutrophils to promote preeclampsia development. Cell Mol Immunol.

[31] Cui J, Kang X, Shan Y, Zhang M, Gao Y, Wu W, Chen L (2022). Mir-1227-3p participates in the development of fetal growth restriction via regulating trophoblast cell proliferation and apoptosis. Sci Rep.

[32] Cowden Dahl KD, Fryer BH, Mack FA, Compernolle V, Maltepe E, Adelman DM, Carmeliet P, Simon MC (2005). Hypoxia-inducible factors 1alpha and 2alpha regulate trophoblast differentiation. Mol Cell Biol.

[33] Kisanga EP, Tang Z, Guller S, Whirledge S (2018). Glucocorticoid signaling regulates cell invasion and migration in the human first-trimester trophoblast cell line Sw.71. Am J Reprod Immunol.

[34] Kasture V, Sundrani D, Randhir K, Wagh G, Joshi S (2021). Placental apoptotic markers are associated with placental morphometry. Placenta.

